# Association of tea and its extracts with colorectal adenomas: meta-analysis and systematic review

**DOI:** 10.3389/fnut.2023.1241848

**Published:** 2023-10-05

**Authors:** Xifei Guan, Nawen Liu, Zhixin Zhu, Yanxue Xu, Dehai Xiong, Xiuyang Li

**Affiliations:** ^1^Department of Big Data in Health Science, and Center for Clinical Big Data and Statistics, The Second Affiliated Hospital, College of Medicine, Zhejiang University, Hangzhou, China; ^2^Department of Nursing, College of Medicine, Zhejiang University, Hangzhou, China; ^3^Department of General Surgery, Three Gorges Affiliated Hospital, Chongqing University, Chongqing, China

**Keywords:** tea, tea extracts, colorectal adenomas, meta-regression, meta-analysis, systematic review

## Abstract

**Background:**

There are many studies on the association of tea and its extracts with colorectal adenomas, but the results have varied. The study aims to investigate the effect of tea and its extracts on colorectal adenomas using meta analysis and systematic review.

**Methods:**

Literature was obtained through PubMed, Cochrane Library, Embase and Chinese BioMedical Literature Service System since the establishment of the database until April 31, 2023. Search terms include adenomas, polyps, colorectal, rectal, rectum, tea, epigallocatechin, drinking and beverages. Meta-regression analysis was used to infer the source of heterogeneity. Heterogeneity was assessed using *I^2^* statistics and *Q* test. The effect measures were odds ratio (OR) and 95% confidence interval (95% CI). Stata17.0 software was used for data processing.

**Results:**

The findings indicated that study design (*t* = 0.78, *P* = 0.454), types of tea intake (*t* = 1.35, *P* = 0.205), occurrences (*t* = -0.19, *P* = 0.852), regions (*t* = 1.13, *P* = 0.281) and grades of adenomas (*t* = 0.06, *P* = 0.952) were statistical homogeneity. Tea and its extracts were negatively correlated with the risk of colorectal adenomas (OR = 0.81, 95% CI: 0.66–0.98). No publication bias was found in this study (*t* = -0.22, *P* = 0.828) and the results are robust.

**Conclusion:**

This study suggests that tea and its extracts have a certain protective effect on colorectal adenomas, which provides scientific evidence for preventive strategies for colorectal adenomas. As for the causal relationship between tea and its extracts on colorectal adenomas, further prospective studies are needed.

## 1. Introduction

Colorectal cancer (CRC) is one of the most common malignancies with high incidence and mortality rates (1). According to the information published by the International Agency for Research on Cancer (IARC) of the World Health Organization, CRC was the third most common cancer in the world after lung cancer and breast cancer in terms of global incidence and mortality in 2020 (2). China’s Guideline for the Screening, Early Detection and Early Treatment of CRC points out that the incidence and mortality rates of CRC are on the rise, causing a serious disease burden in China (3). A recent study showed that the burden of CRC increased significantly worldwide over 30 years and that China, the United States and Japan had the highest number of CRC cases in 2019, with East Asia having the highest burden of CRC (4).

Some studies have found that the development of CRC mostly follows the evolutionary pathway of “adenoma-cancer,” that is, normal mucosa first appears with epithelial hyperplasia-like changes, then can gradually transform into an adenoma, and later can develop into carcinoma in situ and invasive carcinoma, and it generally takes 5–10 years to progress from precancerous lesions to cancer, this provides an important time window for early diagnosis and clinical intervention (5–7). Therefore, the development of CRC can be largely prevented by modifying modifiable risk factors and through the detection and removal of precancerous lesions (8).

Tea is rich in polyphenolic compounds, with catechins as the main component. Studies have shown that catechins have a variety of pharmacological properties, including antioxidant, anti-inflammatory, anti-cancer effects, and so on (9–11). In the gastrointestinal tract, green tea has been found to activate intracellular antioxidants, inhibit the formation of carcinogens, angiogenesis and the proliferation of cancer cells (12, 13).

There are few studies investigating the relationship between tea and the risk of colorectal adenomas. Some studies have found that tea has a negative correlation with the risk of colorectal adenomas and helps reduce the incidence of CRC (14, 15). However, the studies were not in agreement, for example, the results of Chen et al. showed a protective effect of tea consumption on low-risk colorectal adenomas (OR = 0.66, 95% CI: 0.48–0.90) and high-risk colorectal adenomas (OR = 0.57, 95% CI: 0.36–0.89) (16), while the results of Budhathoki et al. found no association between tea intake and the risk of colorectal adenomas (OR = 1.50, 95% CI: 0.97–2.31) (17). Therefore, this study is aimed to analyze and evaluate the relationship between tea and its extracts on colorectal adenomas by using meta-analysis, to provide some scientific evidence for the prevention of colorectal adenomas and CRC through tea consumption.

## 2. Materials and methods

### 2.1. Search strategy

The systematic review and meta-analysis were performed according to the Cochrane Handbook for Systematic Reviews of Interventions (18) and the Preferred Reporting Items for Systematic Reviews and Meta-Analyzes guidelines for meta-analysis (19). This project was registered with the International Prospective Register of Systematic Reviews (PROSPERO) under registration identification number CRD42023396930 (20), and compliant with the main PRISMA statement (21, 22). Studies related to tea and its extracts with colorectal adenomas were identified by searching PubMed, Cochrane Library, Embase and Chinese BioMedical Literature Service System, all from the time of creation to 31 April 2023. Meanwhile, references of included literature were further manually searched to identify additional studies that met the inclusion criteria.

Research terms (adenomas OR polyps) AND (colorectal OR rectal OR rectum) AND (tea OR epigallocatechin OR drinking OR beverages OR diet) were used. The details of research strategy were provided in Supplementary material. Each identified report was carefully scanned by two of the authors.

### 2.2. Selection criteria

Inclusion criteria were as follows: (1) literature investigating the relationship between tea and its extracts with colorectal adenomas, (2) cohort studies, case–control studies, cross-sectional studies, or randomized controlled trials (RCTs), (3) studies reporting the definition of colorectal adenomas and the methods of diagnosis, and (4) literature reporting the sample size of the study, or which could be derived from the original data.

Reports as follows were excluded: (1) literature with repeated reports on the same population, (2) case reports, reviews, letters or comments, (3) animal or cell trials, and (4) literature with incomplete data or where sample sizes for each study result could not be derived from the literature.

### 2.3. Data extraction and quality assessment

Two authors screened the literature independently, extracted information using a uniform data extraction form and cross-checked. In case of disagreement, it was discussed or referred to a third-party evaluation for resolution. Potentially eligible studies were selected by abstracting the title or abstract. If the title or the abstract was inadequate to reach a final decision, the full paper was obtained. Data extraction included: the first author’s name, published year, country, study design, sex ratio, age, cases, sample size, odds ratio (OR) and its 95% confidence interval (95% CI) after adjusting for the most covariates, and adjusted confounders. The Newcastle-Ottawa scale was used to assess the quality and risk of bias of observational studies (23), and studies scoring seven or more than seven were defined as high-quality studies. The Cochrane Collaboration’s tool was used to assess the quality of RCTs (24).

### 2.4. Statistical analysis

Observational studies collected effect sizes for each study, using the OR and its 95% CI as the effect analysis statistics, and clinical trials performed quantitative pooled analyzes, calculating combined estimates expressed as relative risk (RR) with corresponding 95% CI to test the association between tea and its extracts on the risk of colorectal adenomas. Heterogeneity between studies was assessed using Cochran’s *Q* test and the *I^2^* statistic (25, 26). Heterogeneity was considered to be significant if *I^2^* > 50% and *P* ≤ 0.05, and analysis was performed using a random-effect model, conversely, there was no heterogeneity and a fixed-effect mode can be used. Forest plots were produced to show the results of each trial and estimate the overall effect size. Publication bias was assessed using funnel plots and Egger’s test (27). Sensitivity analysis was conducted by omitting one estimate at a time sequentially and recalculating the pooled results to measure the effect of each study on the overall effects and the robustness of the results.

All statistical analyzes were performed by STATA software (Version 17.0, Stata Corporation, College Station, TX, United States). The differences were considered to be statistically significant at *P*-value ≤0.05 with the two-sided test.

## 3. Results

### 3.1. Search results and study characteristics

**Figure 1:**
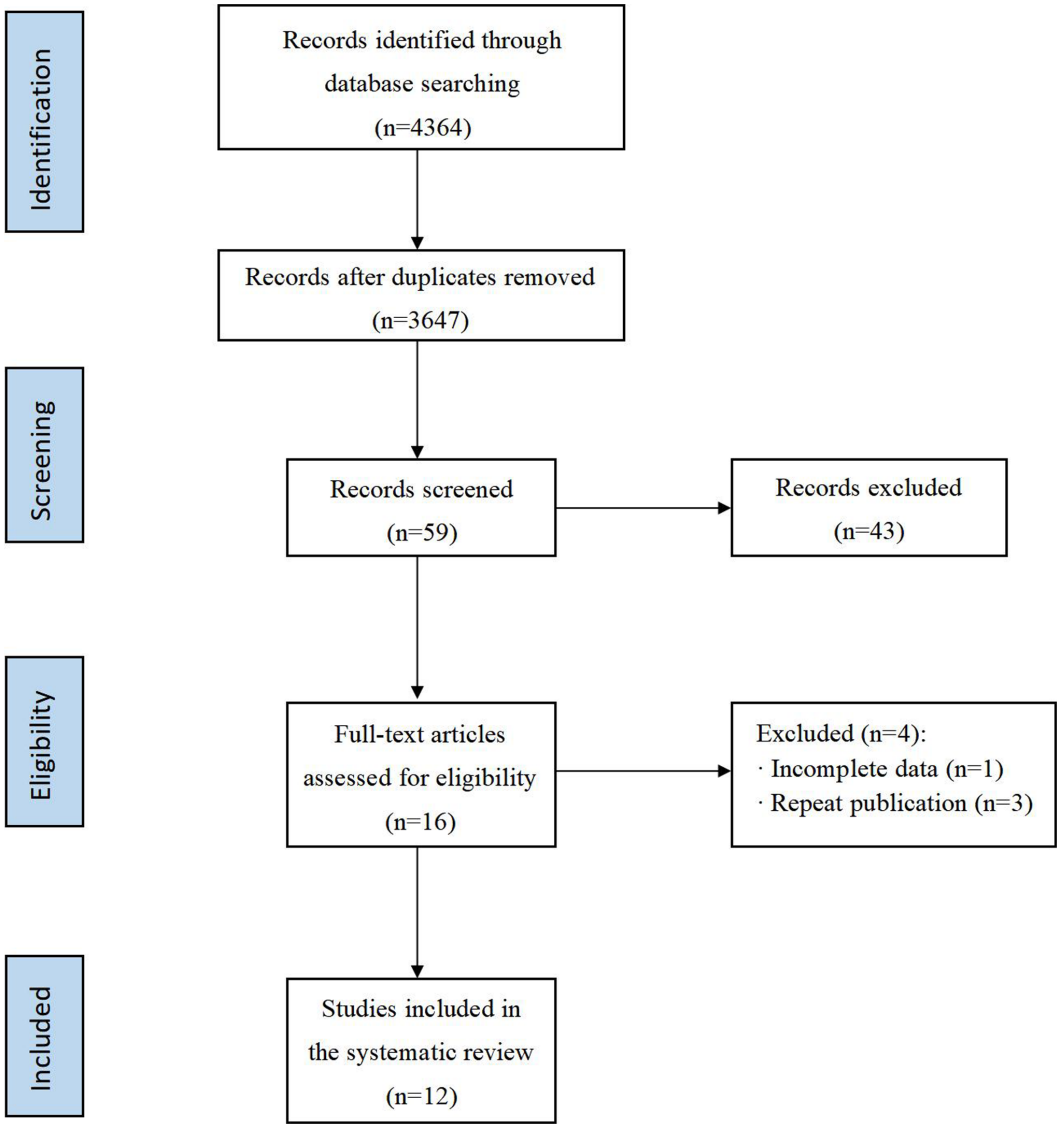
PRISMA flow diagram of the study selection process.

The literature screening process was shown in Figure 1. In the initial screening process, a total of 4,364 articles were retrieved from the database, after removing duplicate articles, the titles and abstracts of the remaining 3,647 articles were read, and 59 articles were retained after excluding those that did not meet the study criteria. After a full-text review, 47 papers were excluded, and a total of 12 papers were finally included for systematic review and meta-analysis (16, 17, 28–37).

The basic characteristics of the included studies were shown in Table 1. All articles were published between 1990 and 2022, including one cross-sectional study, two cohort studies, five case–control studies, and four RCTs. The risk of bias assessment for the observational studies and RCTs was presented in Supplementary Table S1 and Supplementary Figure S1, respectively. According to the Newcastle-Ottawa scale and the Cochrane collaboration’s tool, the quality of the observational studies was high, and there was no significant risk of bias in the included RCTs.

**Table 1:** Characteristics of included studies in the meta-analysis.

Study, year	Location	Study design^a^	Age^b^	Males/Females	Occurrence	Cases	Control/sample size	Type of tea intake	Factors/Comparison (highest v.lowest)	Results	Adjusted confounders
Seufferlein, 2022	Germany	RCT	45–80	406/226	Recurrence	338	632	EGCG	300 mg/day v. placebo	RR = 0.92 (95% CI: 0.79–1.06)	Age, low-dose aspirin, center
Chen, 2021	China	Cross-sectional study	50.26 ± 11.95	4111/2907	Incidence	670	7,018	Green tea	≥42 cup-year v. none	OR = 0.63 (95% CI: 0.49–0.82)	Age, sex, obesity, smoking, alcohol use, hypertension, diabetes, cholesterol/high-density cholesterol, NSAID use, family history of CRC, regular exercise
Sinicrope, 2021	USA	RCT	61.7 ± 8.6	22/10	Recurrence	10	31	EGCG	390 mg/day v. placebo	RR = 0.81 (95% CI: 0.28–2.31)	Age, sex, race, center, history of disease, aspirin use
Shin, 2018	Korea	RCT	19–85	97/46	Recurrence	47	143	EGCG	206 mg/day v. placebo	RR = 0.56 (95% CI: 0.34–0.92)	Age, sex, BMI, smoking, alcohol drinking, comorbidities, laboratory findings, baseline colonoscopy findings
Nakamura, 2016	Japan	Cohort study	40–65	297/0	Recurrence	164	297	Green tea	>6cup/day v. 0cup/day	OR = 0.68 (95% CI: 0.26–1.74)	Age, BMI, physical activity, alcohol consumption, current smoking status, energy intake, dietary fat, dietary fiber, caffeine consumption, fasting serum glucose, and the randomization group from the previous study
Budhathoki, 2015	Japan	Case–control study	40–79	951/484	Incidence	738	697	Green tea	>960.0 mL/day v. <194.3 mL/day	OR = 1.50 (95% CI: 0.97–2.31)	Age, sex, BMI, smoking, alcohol drinking, screening period, physical activity, parental CRC history, history of diabetes mellitus, NSAID use, total energy, folate, fiber, isoflavone, red and processed meat intake
Shimizul, 2008	Japan	RCT	20–80	81/44	Recurrence	29	125	EGCG	157.5 mg/day v. 0 mg/day	RR = 0.49 (95% CI: 0.24–0.99)	Age, sex, tea consumption, number, size and histological grading of initial adenomas
Il’yasova, 2003	USA	Case–control study	30–80	196/298	Incidence	171	323	Black tea	>3servings/day v. <2servings/day	OR = 0.50 (95% CI: 0.20–1.10)	Age, sex, BMI, race, apoptotic score
Baron, 1997	USA	Cohort study	61.3 ± 8.3	520/146	Recurrence	243	666	Black tea	≥1cup/day v. 0cup/day	OR = 1.29 (95% CI: 0.75–2.22)	Age, sex, smoking, alcohol intake, center, colonoscopy interval, total caloric intake, dietary fat, dietary fiber
Olsen, 1993	Denmark	Case–control study	45–74	214/319	Incidence	171	362	Black tea	≥4 cup/day v. 0–3 cup/day	OR = 1.30 (95% CI: 0.70–2.30)	Age, sex, dietary fiber
Kono, 1991	Japan	Case–control study	49–56	1228/0	Incidence	80	1,148	Green tea	≥5 cups/day v. <3 cups/day	OR = 0.69 (95% CI: 0.61–1.95)	Smoking, alcohol drinking, physical activity
Kato, 1990	Japan	Case–control study	35–80	716/387	Incidence	525	578	Green tea and Black tea	≥1cup/day v. <1cup/day	Green tea: OR = 0.63 (95% CI: 0.50–0.79); Black tea: OR = 0.95 (95% CI: 0.55–1.63)	Age, sex, residence

a If studies reported results from the same population and their investigated factors overlapped, the most recent publications or studies with the biggest sample size were used for meta-analysis.

b The age variable was described in terms of mean (standard deviation) or age range.

BMI, body mass index; CRC, colorectal cancer; EGCG, epigallocatechin gallate; NSAID, non-steroidal anti-inflammatory drug; RCT, randomized controlled trial.

### 3.2. Effects of tea and its extracts on the colorectal adenomas

**Figure 2:**
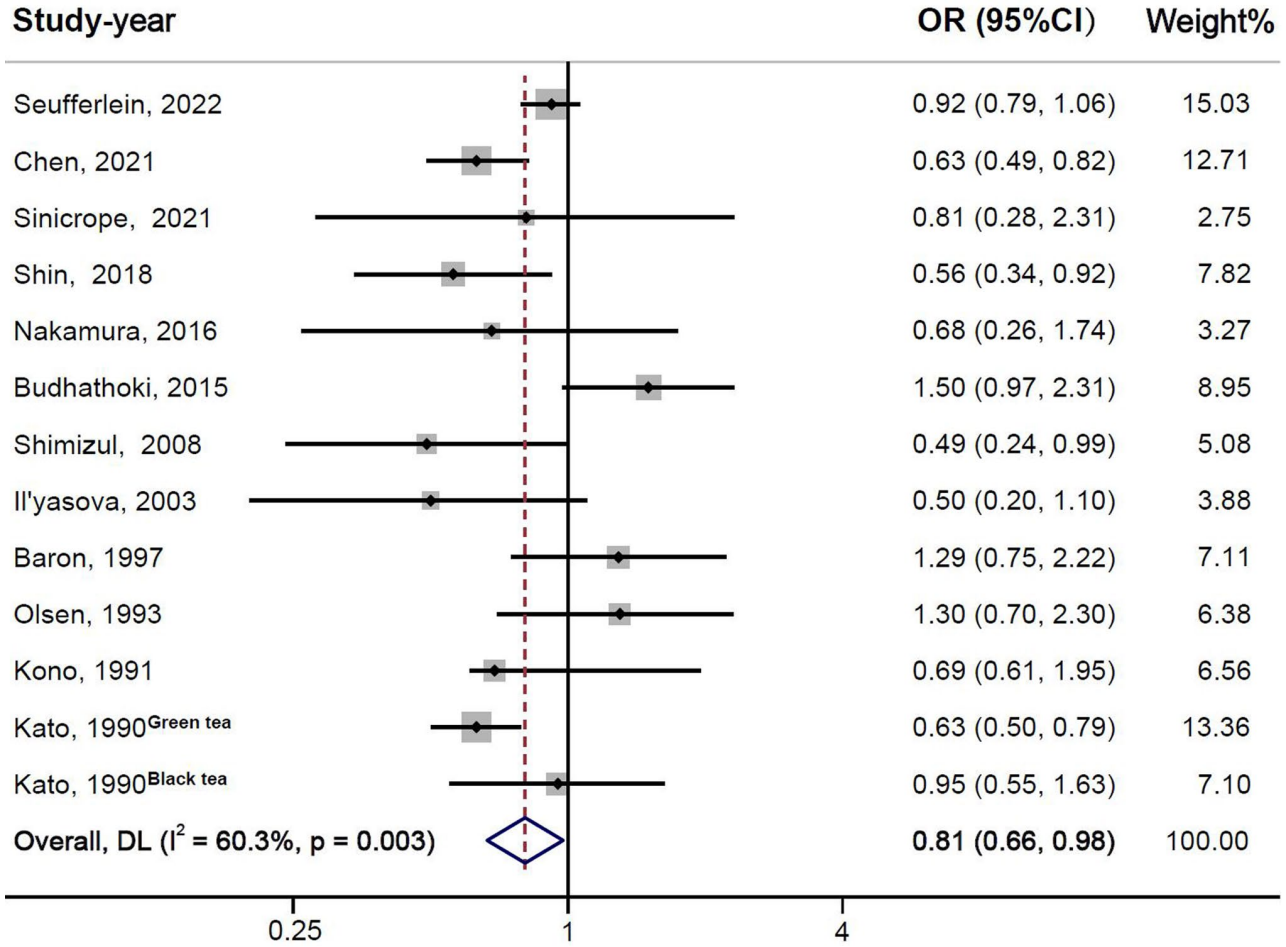
Forest plot of tea and its extracts with colorectal adenomas.

The results of the overall meta-analysis were presented in Figure 2. The heterogeneity test showed the existence of heterogeneity between studies (*I^2^* = 60.3%, *P* = 0.003), so a random-effect model was used for the analysis. The results indicated that tea and its extracts could reduce the risk of colorectal adenomas (OR = 0.81, 95% CI: 0.66–0.98).

### 3.3. Meta-regression and subgroup analysis

Meta-regression analysis was conducted according to two study design types, observational studies and clinical trials, two types of tea intake, green and black tea, two occurrences, incidence and recurrence, three regions, East Asia, North America, and Europe, and two grades of adenomas, low and high. The results were presented in Table 2, which showed that there was no heterogeneity between study design types, types of tea intake, occurrences, regions and grades of adenomas.

**Table 2:** Results of univariate meta-regression analysis.

Covariates	Coefficient	SE	*t*-value	*P*-value
Study design	0.1860	0.2397	0.78	0.454
Type of tea intake	0.3099	0.2302	1.35	0.205
Occurrence	−0.0427	0.2233	−0.19	0.852
Region	0.1545	0.1361	1.13	0.281
Grade of adenomas	0.0165	0.2581	0.06	0.952

The results of the subgroup analysis were presented in Supplementary Table S2 and Supplementary Figure S2. The association was significant in green tea (OR = 0.75, 95% CI: 0.60–0.93), while there was no association between black tea and colorectal adenomas (OR = 1.03, 95% CI: 0.72–1.47). Subgroup analysis based on region indicated that tea and its extracts reduced the risk of colorectal adenomas by 27% (95% CI: 0.57–0.92) in East Asia, while results showed that tea and its extracts were not associated with colorectal adenomas in North America (OR = 0.87, 95% CI: 0.48–1.59) and Europe (OR = 0.97, 95% CI: 0.76–1.22). The association was more significant between tea and its extracts with low-risk colorectal adenomas (OR = 0.74, 95% CI: 0.57–0.95), but not with the risk of high-risk colorectal adenomas (OR = 0.73, 95% CI: 0.47–1.14).

### 3.4. Publication bias and sensitivity analysis

**Figure 3:**
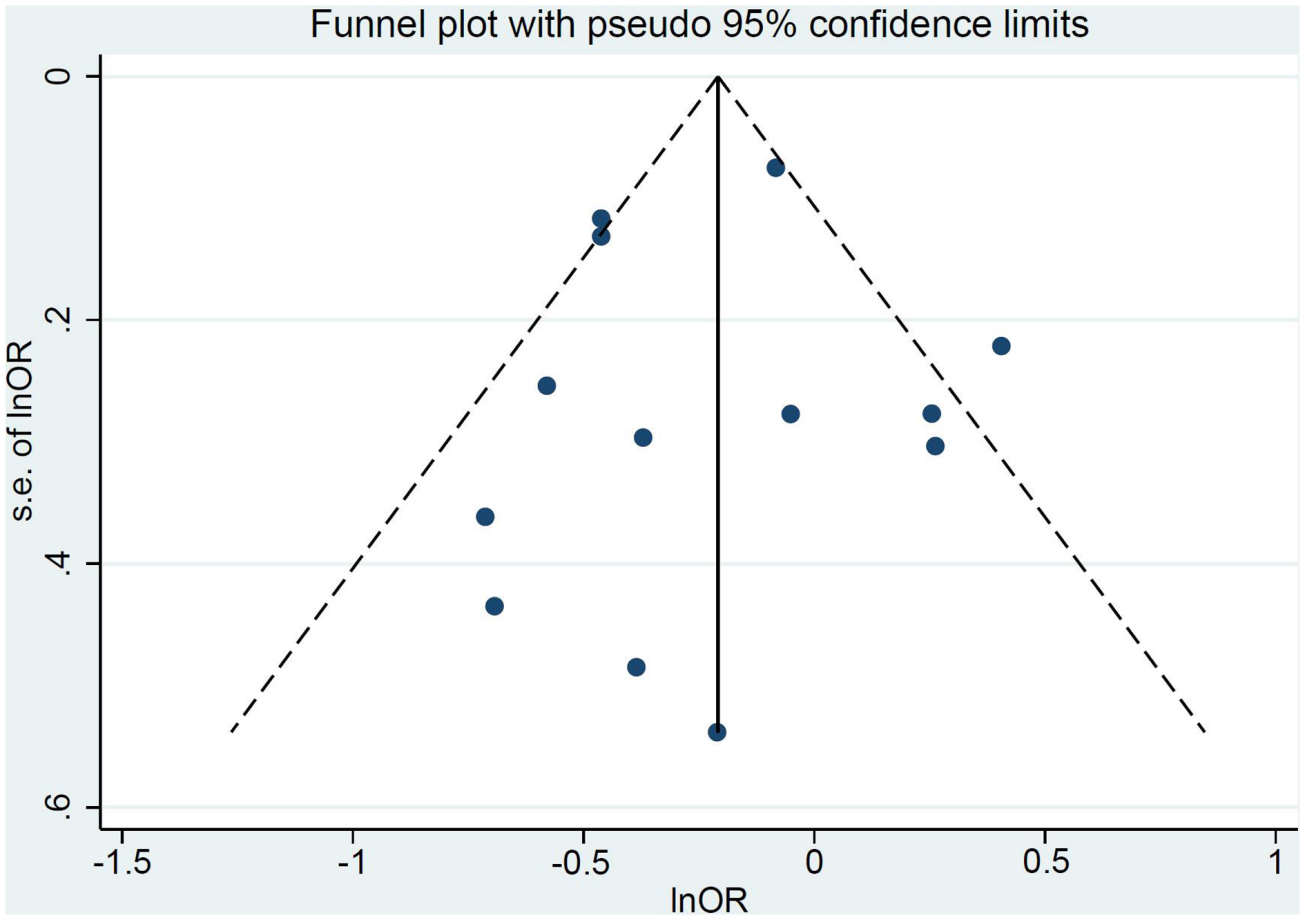
The funnel plot of included studies.

**Figure 4:**
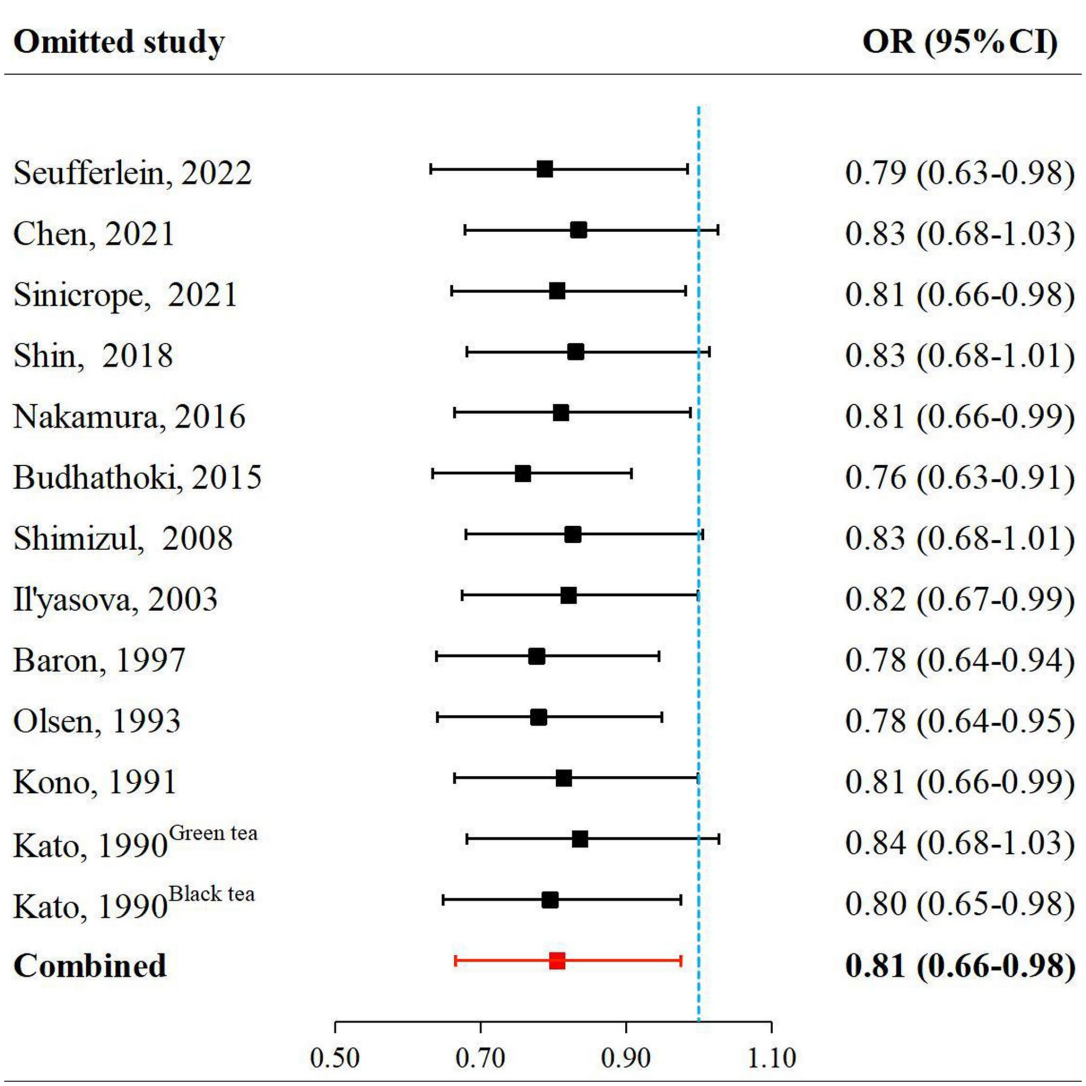
Sensitivity analyzes by omitting one study at a time.

Funnel plots of the studies were shown in Figure 3. Visual inspection was largely symmetrical, and the results of Egger’s test also showed that there was no publication bias (*t* = −0.22, *P* = 0.828). The results of sensitivity analysis showed no impact of excluding any single study on the overall estimate of the effect of tea and its extracts on the risk of colorectal adenomas, indicating that the analysis was robust (Figure 4; Table 3).

**Table 3:** The results of sensitivity analyzes.

Study omitted	OR	95% CI
Seufferlein, 2022	0.79	0.63–0.98
Chen, 2021	0.83	0.68–1.03
Sinicrope, 2021	0.81	0.66–0.98
Shin, 2018	0.83	0.68–1.01
Nakamura, 2016	0.81	0.66–0.99
Budhathoki, 2015	0.76	0.63–0.91
Shimizul, 2008	0.83	0.68–1.01
Il’yasova, 2003	0.82	0.67–0.99
Baron, 1997	0.78	0.64–0.94
Olsen, 1993	0.78	0.64–0.95
Kono, 1991	0.81	0.66–0.99
Kato, 1990^Green tea^	0.84	0.68–1.03
Kato, 1990^Black tea^	0.80	0.65–0.98
Combined	0.81	0.66–0.98

## 4. Discussion

This meta-analysis and systematic review is the latest study investigating tea and its extracts and their influence on the risk of colorectal adenomas. The meta-analysis of 12 studies indicates that tea and tea extract supplements have a protective effect against colorectal adenomas.

Tea originated from the plant species *Camellia sinensis* and has become the second most commonly consumed beverage in the world, with green tea, black tea and oolong tea being among the beverages often consumed (38). Tea is rich in many active ingredients, the main component of polyphenols, which has been proven in animal and cellular experiments to inhibit the formation and proliferation of CRC and other tumors (39, 40). Different types of tea contain different types and contents of polyphenols, theaflavin-3, 30-digallate (TFDG) in black tea and epigallocatechin-3-O-gallate (EGCG) in green tea have a protective effect against oxidative stress, these substances are involved in many biochemical processes responsible for the inflammation and proliferation of cancer cells, all of which reduce the phenomenon and symptoms of intestinal inflammation, reduce the proliferation of intestinal polyps and adenomas and reduce the proliferation of CRC cells (41–44).

Chen et al. (45) reported a meta-analysis on the association between tea consumption and CRC, which enrolled cohort studies and case–control studies. The results among all studies showed that compared with the lowest tea consumption, the highest tea consumption reduced the risk of CRC by 7% (OR = 0.93, 95% CI: 0.87–1.00), indicating that tea consumption had an effect on the prevention of colorectal tumors, which was consistent with the results of our study. However, a meta-analysis of case–control studies showed that high-dose green tea intake may marginally reduce the risk of colorectal tumors, without statistical significance (OR = 0.95, 95% CI: 0.81–1.11) (46); another meta-analysis based on prospective cohort studies also showed no significant association between tea consumption and colorectal tumor risk (RR = 0.97, 95% CI: 0.94–1.01) (47), which was similar to the results of the former study.

The results of subgroup analyzes showed a protective effect of green tea and its extracts against colorectal adenomas, as well as in East Asian populations. This may be due to the prevalence of tea culture in Asia, where the content and frequency of tea drinking are much higher than those in North America and Europe. Moreover, green tea is mostly consumed in Asia, while black tea is mostly consumed in North America and Europe. This result is consistent with the results of the subgroup analysis of tea intake types in the previous section. Chen et al. also reported that significant correlations were observed in the green tea and CRC in their meta-analysis (45), which was similar to our findings. In a meta-analysis based on case–control studies, Wang et al. (46) found that tea had an insignificant protective effect against colorectal tumors in Asia (OR = 0.87, 95% CI: 0.62–1.22) in addition to the US and European populations, whereas the results of our study showed that tea had a pronounced protective effect against colorectal adenomas in East Asian populations. In terms of adenomas grade, the results showed that tea and its extracts reduced the risk of low-risk colorectal adenomas (OR = 0.74, 95% CI: 0.57–0.95), but were not correlated with the risk of high-risk colorectal adenomas (OR = 0.73, 95% CI: 0.47–1.14). However, only three of the included studies graded the adenomas, so the results may be biased. Chen et al. showed that tea consumption ≥42 cup-years reduced the incidence of low-risk adenomas (OR = 0.66, 95% CI: 0.48–0.90) and high-risk adenomas (OR = 0.57, 95% CI: 0.36–0.89), but when tea consumption <42 cup-years, it only had a protective effect against low-risk adenomas, not between high-risk adenomas (16). Meanwhile, Nakachi et al. (48) found that the incidence of CRC was lower among patients who consumed more than 10 cups of green tea per day. Since no significant differences were observed in subjects who consumed less than 10 cups per day, there may be a threshold amount of tea protection, and the threshold for high-risk adenomas was higher than that for low-risk adenomas. Other factors such as subject selection bias, diet, alcohol consumption, smoking (49), type of tea, total tea consumption, measurement error (50), and temperature of tea infusion may also affect the study results.

The strength of this study is that we enrolled all relevant studies (*n* = 12), with a larger proportion of RCT studies and cohort studies, and a higher level of confidence in the results of the analysis. Besides, we performed relatively comprehensive analyzes including meta-regression analysis, subgroup analysis, publication bias detection, and sensitivity analysis to explore potential sources of heterogeneity and bias.

However, our study still has several limitations. Firstly, the number of enrolled studies was limited, so the number of literature on the subgroup analysis groups was fewer, and their results may be unstable. Secondly, there is the issue of measurement error, which may result in bias in the calculation of estimated effects and the judgment of confounding factors due to different methods of measuring the dosage of tea and its extracts included in the study. Finally, because tea is mainly prevalent in Asia, the relatively large sample size of Asians in this study leads to a potential selection bias, caution should be exercised when extrapolating the results of this study to other countries. More studies are needed to confirm the preventive effect of tea on the risk of colorectal adenomas, especially for populations of different genders in different regions.

## 5. Conclusion

The result from the meta-analysis of 12 studies indicates that tea and its extract supplements have a protective effect against colorectal adenomas. Subgroup analyzes show that the protective effects of tea and its extracts against colorectal adenomas are more pronounced in green tea, East Asian populations, and low-risk adenomas, but the number of literature on the subgroup analysis groups was fewer, which needs to be determined by more evidence. The findings may provide a theoretical basis for the development of nutrition guidelines. As for the causal relationship between tea and its extracts on colorectal adenomas, further prospective studies are needed.

## Author contributions

XL, DX, and XG conceived and designed the meta-analysis. XG, NL, ZZ, and YX searched the literature. XG, NL, and ZZ extracted the data. XG analyzed the data. ZZ contributed analysis tools. XG and NL wrote the manuscript. XL revised the manuscript. All authors contributed to the article and approved the submitted version.

## Abbreviations

CI: Confidence interval
CRC: Colorectal cancer
EGCG: Epigallocatechin-3-O-gallate
IARC: International Agency for Research on Cancer
OR: Odds ratio
RCTs: Randomized controlled trials
RR: Relative risk
TFDG: Theaflavin-3, 30-digallate
