# Supplementary material for: Association of tea and its extracts with colorectal adenomas: meta-analysis and systematic review
Source: Front Nutr. 2023 Oct 5;10:1241848. doi: 10.3389/fnut.2023.1241848 (PMC10585173; doi:10.3389/fnut.2023.1241848)
Supplement: Supplementary file 1 [file Data_Sheet_1.pdf]

Supplementary Material

Association of Tea and Its Extracts with Colorectal Adenomas: Meta-analysis and Systematic Review

Xifei Guan, Nawen Liu, Zhixin Zhu, Yanxue Xu, Dehai Xiong*, Xiuyang Li*

*** Correspondence:** Xiuyang Li: lixiuyang@zju.edu.cn; Dehai Xiong: 1208505185@qq.com.

Supplementary Table 1. Summary of Newcastle Ottawa Scale* scores for observational studies.

Study, year	Selection (maximum 4 marks)	Comparability (maximum 2 marks)	Outcome/Exposure (maximum 3 marks)	Total (maximum 9 marks)
Chen, 2021	4	2	2	8
Nakamura, 2016	4	2	2	8
Budhathoki, 2015	4	2	2	8
Il'yasova, 2003	4	1	2	7
Baron, 1997	4	1	3	8
Olsen, 1993	3	1	3	7
Kono, 1991	4	1	2	7
Kato, 1990	4	1	2	7

*Categories for Case-control studies include: Selection (adequate colorectal adenoma case definition, colorectal adenoma case representativeness, control selection, non-colorectal adenoma control definition), Comparability (differences between groups in age and sex factors), and Exposure (ascertainment of risk factor, similar method of risk factor ascertainment for cases and controls, and response rates). Categories for Cohort studies include: Selection (risk factor-exposed cohort representativeness, cohort selection, ascertainment of risk factors, evidence of no colorectal adenoma at the start of the study), Comparability (differences between groups in age and sex factors), and Outcome (colorectal adenoma assessment, adequate follow-up length for colorectal adenoma detection, and adequacy of cohort follow-up).

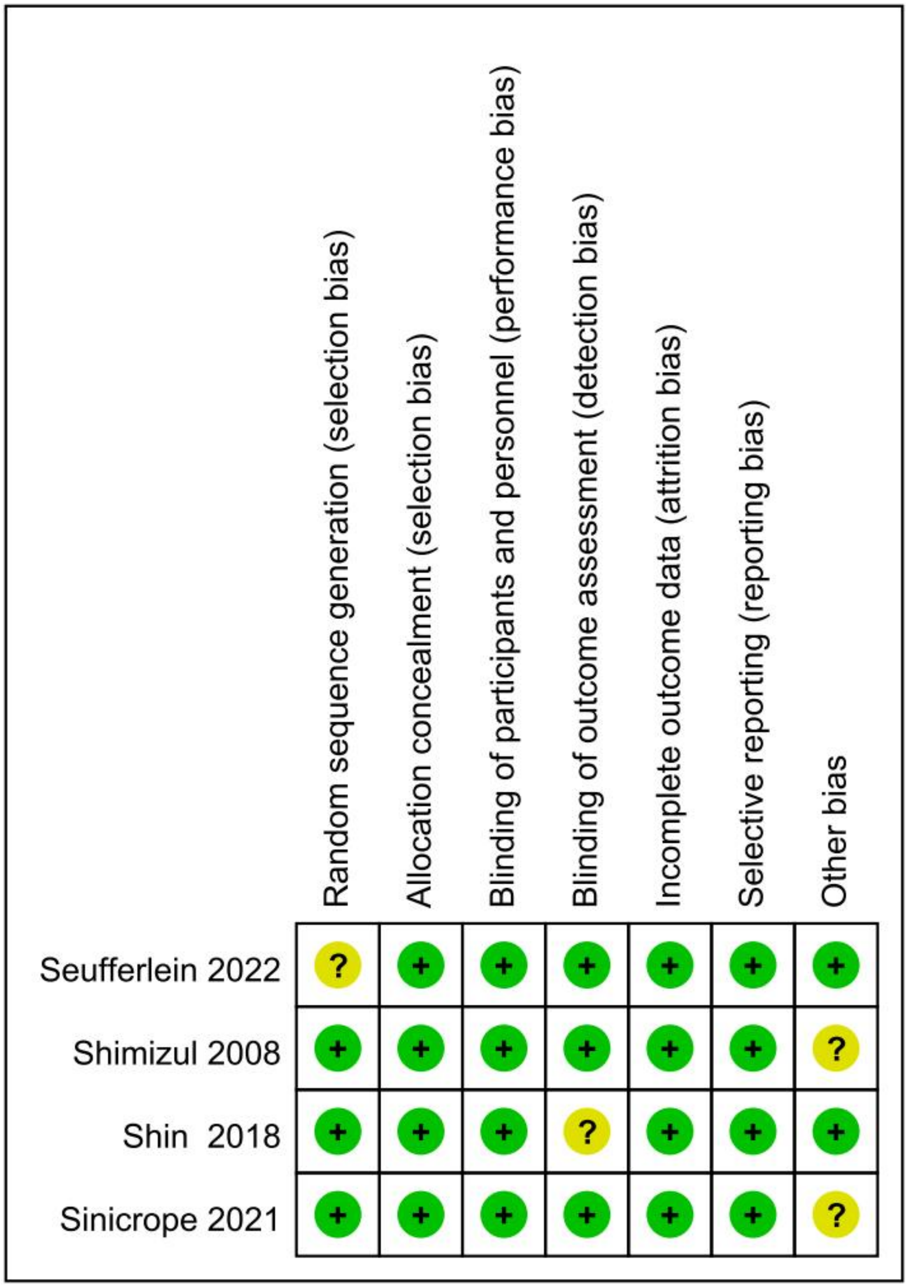

Supplementary Figure 1. The risk of bias assessment for RCTs.

Supplementary Table 2. Subgroup analysis of type of tea intake, occurrence, region and grade of adenomas.

Subgroup	Number	OR (95% CI)	Heterogeneity	
			I^2 (%)	*P*
**Overall effect**	13	**0.81 (0.66, 0.98)**	60.30	0.003
**Type of tea intake**				
Green tea	9	**0.75 (0.60, 0.93)**	65.40	0.003
Black tea	4	1.03 (0.72, 1.47)	26.60	0.252
**Occurrence**				
Incidence	7	0.82 (0.62, 1.09)	67.90	0.005
Recurrence	6	0.80 (0.61, 1.06)	40.20	0.138
**Region**				
East Asia	8	**0.73 (0.57, 0.92)**	57.00	0.023
North America	3	0.87 (0.48, 1.59)	42.60	0.175
Europe	2	0.97 (0.76, 1.22)	18.30	0.269
**Grade of adenomas**				
Low	3	0.74 (0.57-0.95)	37.50	0.202
High	3	0.73 (0.47-1.14)	33.10	0.225

*The between-subgroup heterogeneity was assessed using univariate meta-regression analysis. Bold indicates statistical significance.

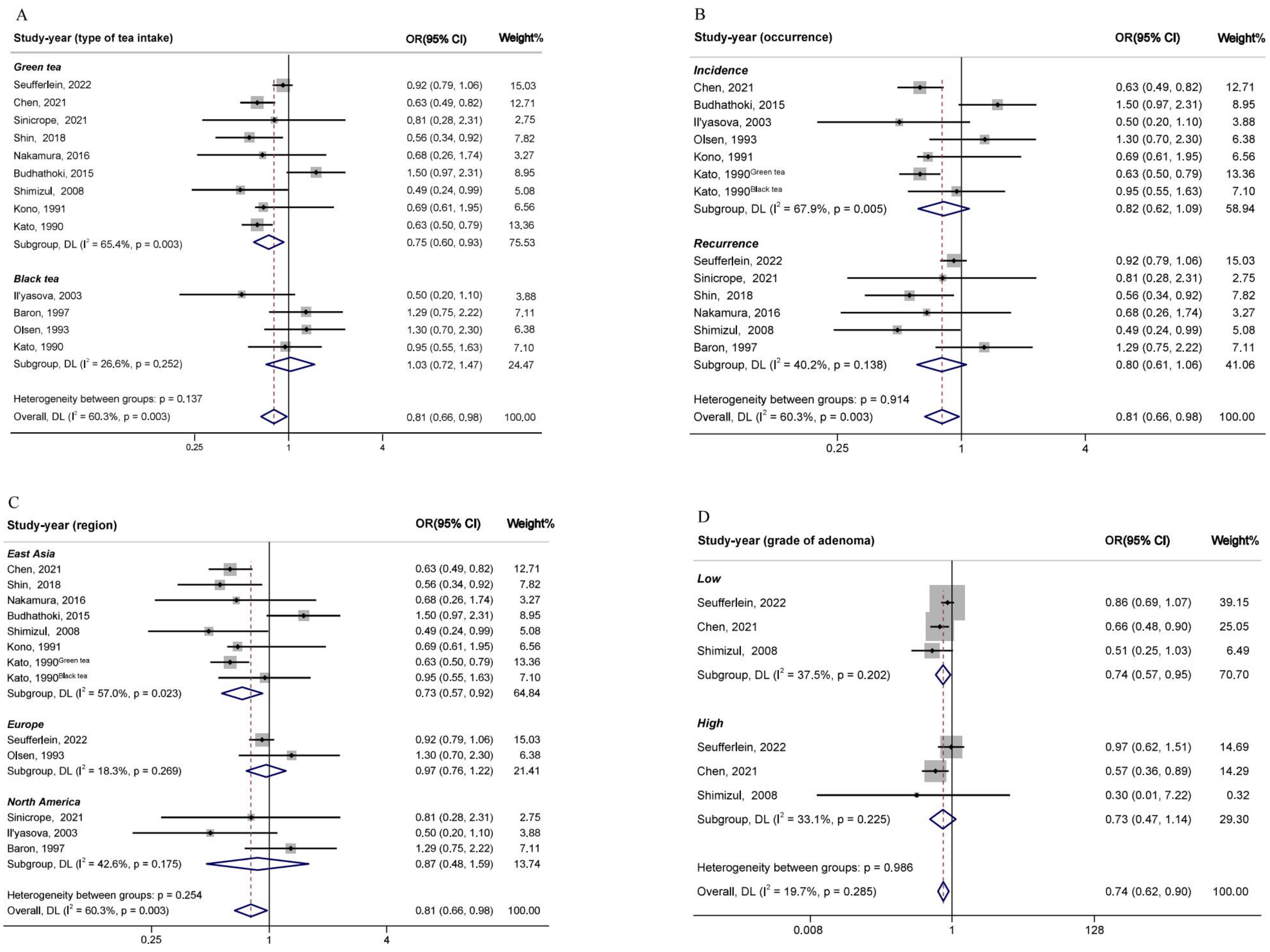

Supplementary Figure 2. The results of subgroup analyses by (A) type of tea intake, (B) occurrence, (C) region, and (D) grade of adenomas.

Search Strategy

Pubmed:
((drinking) OR (beverages) OR (diet) OR (tea) OR (epigallocatechin)) AND (((("Adenomatous Polyps"[MeSH Terms] OR "Adenoma"[MeSH Terms] OR "Adenoma"[Text Word] OR "adenomas"[Text Word] OR "adenomatous"[Text Word] OR "Polyps"[MeSH Terms:noexp] OR "Intestinal Polyps"[MeSH Terms] OR "polyp"[Text Word] OR "Polyps"[Text Word]) AND ("intestine, large"[MeSH Terms] OR "Colorectal Neoplasms"[MeSH Terms:noexp] OR "Colonic Neoplasms"[MeSH Terms:noexp] OR "Rectal Neoplasms"[MeSH Terms:noexp] OR "colon"[Text Word] OR "rectum"[Text Word] OR "colonic"[Text Word] OR "rectal"[Text Word] OR "colorectal"[Text Word] OR "colorectal"[Text Word]))))

Embase:
('large intestine tumor'/exp OR 'colon'/exp OR 'rectum'/exp OR colon:ab,ti OR rectum:ab,ti OR colonic:ab,ti OR rectal:ab,ti OR colorectal:ab,ti OR 'colorectal':ab,ti) AND 'risk' AND ((adenoma:ab,ti OR adeonmas:ab,ti OR adenomatous:ab,ti OR polyp*:ab,ti) OR 'colorectal adenoma'/exp OR 'intestine polyp'/exp OR 'adenoma'/exp) AND ('tea' OR 'epigallocatechin' OR 'drinking' OR 'beverages' OR 'diet')

Cochrane：

#1 MeSH descriptor: [Polyps] explode all trees

#2 MeSH descriptor: [Adenoma] explode all trees

#3 (Adenomatous Polyps OR Adenoma OR adenomas OR adenomatous OR "Polyps OR Intestinal Polyps OR "polyp) (Word variations have been searched)

#4 (drinking) OR (beverages) OR (diet) OR (tea) OR (epigallocatechin) (Word variations have been searched)

#5 #1 or #2 or #3

#6 ((colorectal OR colorectum OR colon* OR rectal OR rectum)) (Word variations have been searched)

#7 #4 and #5 and #6
